# Extracting novel hypotheses and findings from RNA-seq data

**DOI:** 10.1093/femsyr/foaa007

**Published:** 2020-02-03

**Authors:** Tyler Doughty, Eduard Kerkhoven

**Affiliations:** Department of Biology and Biological Engineering, Chalmers University of Technology, Kemivägen 10, 41296, Gothenburg, Sweden

**Keywords:** yeast, transcriptome, RNA-seq data analysis, lncRNA, phylostratigraphy, GO term analysis

## Abstract

Over the past decade, improvements in technology and methods have enabled rapid and relatively inexpensive generation of high-quality RNA-seq datasets. These datasets have been used to characterize gene expression for several yeast species and have provided systems-level insights for basic biology, biotechnology and medicine. Herein, we discuss new techniques that have emerged and existing techniques that enable analysts to extract information from multifactorial yeast RNA-seq datasets. Ultimately, this minireview seeks to inspire readers to query datasets, whether previously published or freshly obtained, with creative and diverse methods to discover and support novel hypotheses.

## Teaser

This review describes how advances in scientific understanding during recent years can inform modern big data analyses of yeast biology and biotechnology.

## INTRODUCTION

**Figure 1.**
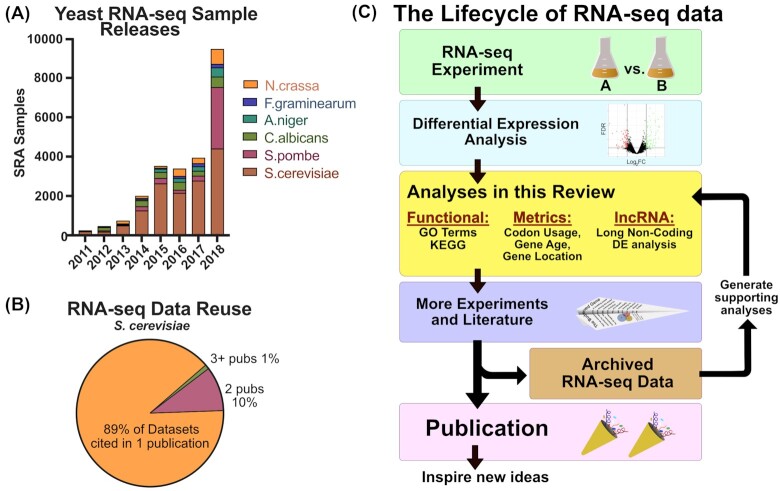
RNA-seq data is increasingly commonly in yeast biology. **(A)** The sequence read archive ‘Run Selector’ tool was used to identify RNA-seq samples uploaded for the yeast species listed. **(B)** RNAseq dataset identifiers acquired from the Sequence Read Archive (SRA) were queried using google scholar and pubmed to assess the number of times a dataset was analyzed in unique peer-reviewed publications. Only published datasets released before January 2017 were included (n = 204). **(C)** A flow diagram showing a frequent paradigm of RNA-seq dataset creation/usage (left). The topics covered in this review are highlighted in yellow.

Next-generation sequencing of the whole transcriptome, or RNA-seq, has been used to further biological understanding of organisms for over a decade (Bainbridge *et al*. 2006). In the interim, RNA-seq has become an increasingly popular method for understanding transcriptome-wide changes in various model systems. The popularization of this type of data analysis has spurred the development of open access programs for quality control, mapping and differential expression (DE) analysis (reviewed in Chowdhury, Bhattacharyya and Kalita 2018). Improvements in the efficiency of these tools coupled with the small gene set size of most yeast species enables small to medium scale RNA-seq datasets to be processed and analyzed using a laptop. Furthermore, published data is increasingly uploaded to publicly available archives (Fig. 1A), and can be downloaded by users in raw format (https://www.ncbi.nlm.nih.gov/sra). These factors are lowering the barrier to analysis and are enabling more scientists, with more diverse scientific backgrounds, to create and test hypotheses with RNA-seq data. While the main theme of this work is how to get more out of ‘your’ dataset, we note that analyses from each section are also opportunities to extract additional information from archived data. Furthermore, we note that despite readily available tools for ensuring dataset quality (Conesa *et al*. 2016), few archived data were found to be the subject of reanalysis (Fig. 1B), which is an opportunity for analysts to gain new insights prior to or in the place of them generating their own RNA-seq datasets.

The sections of this review discuss RNA-seq analyses using (i) gene function (GO Terms), (ii) quantifiable gene metrics (like the evolutionary age of genes) and (iii) non-protein coding RNAs (long non-coding RNAs). Each of these analyses combine DE analysis results (described in chapter 1 of Marchi *et al*. 2017) with additional information (e.g. gene functional information) to help explain transcriptome-wide trends (Fig. 1C). Each section discusses how over the past few years, changes in analysis tools, analysis concepts and areas of interest open up new opportunities for expanding scientific knowledge.

## SECTION 1: THE EMERGENCE OF NEW FUNCTIONAL INFORMATION INFLUENCES GO ENRICHMENT RESULTS

**Figure 2.**
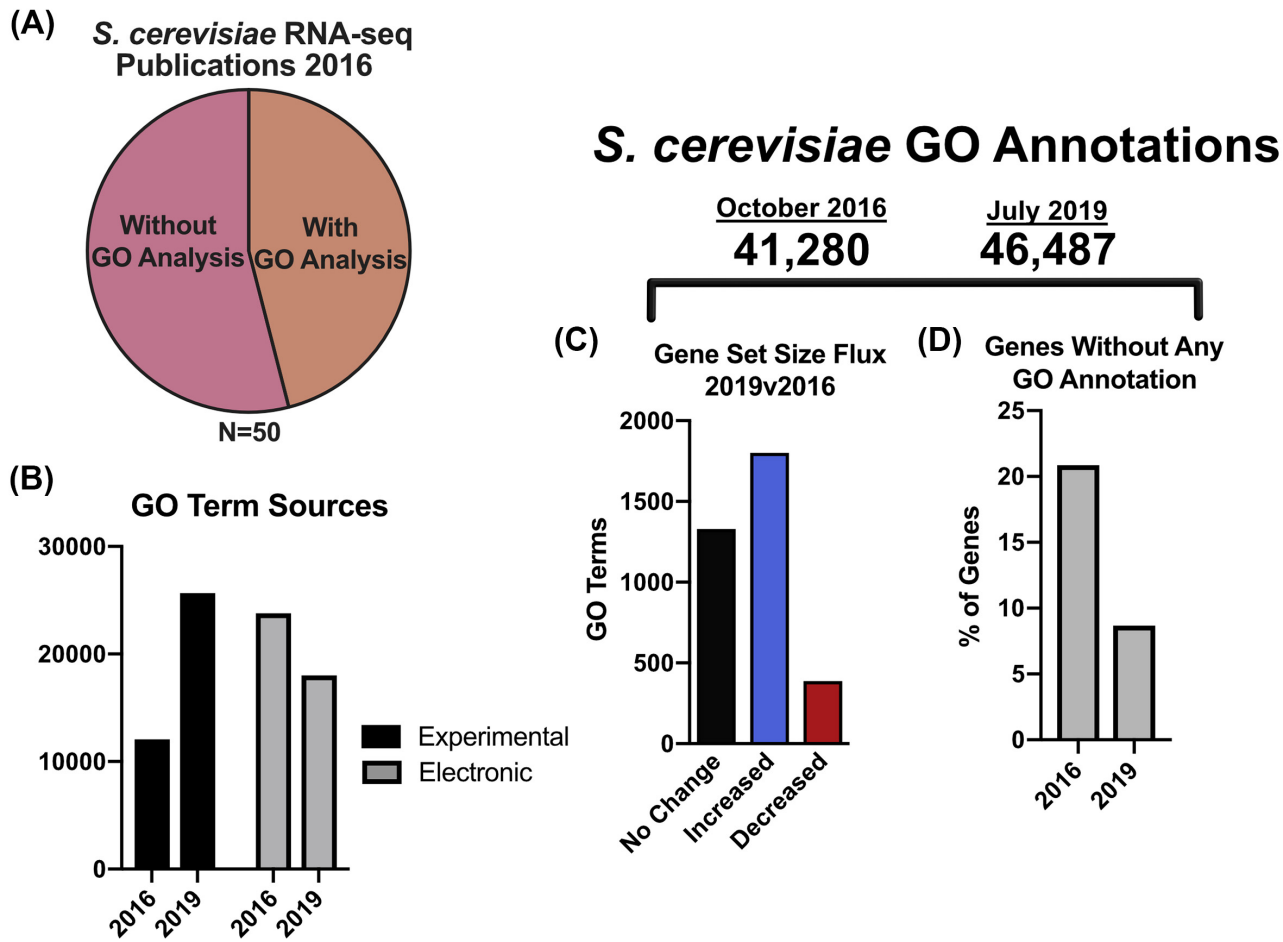
*Saccharomyces cerevisiae* GO terms analyses are common and GO term annotations are regularly updated. **(A)** 50 publications with RNA-seq data from 2016 were manually surveyed for the presence of analyses of GO term enrichment. B–D GO terms ascribed to *S. cerevisiae* genes were obtained from Ensembl in 2019 and Ensembl October 2016 (archive), these were compared to assess GO term source **(B)**, gene set size changes **(C)** and to identify the number of genes lacking any GO term annotation **(D)**.

RNA-seq is a valuable tool for measuring expression changes for transcripts in response to an experimental condition, like gene deletion or a change in environment. To test these changes for significance between replicates, analysts often employ DE analysis, which can result in hundreds of significant gene expression changes, which makes deciphering changes in cellular function challenging. One common method that is used as a starting point to understand systematic changes present in transcriptome data is Gene Set Enrichment Analysis (GSEA) (Subramanian *et al*. 2005). GSEA is used to query DE data for sets of genes that exhibit statistically significant expression changes in experimental samples. One use of GSEA is to assess the enrichment of Gene Ontology terms (or GO terms), which are descriptors for the biological processes, molecular functions and cellular components for each gene (Christie, Hong and Cherry 2009). For each GO term (e.g. Histidine Biosynthesis genes), GSEA assesses whether the expression changes are significant compared to all gene expression changes. Analyses using GSEA to assess GO term enrichment appeared in 46% of the *Saccharomyces cerevisiae* RNA-seq publications surveyed in this work (Fig. 2A). The prevalence of GO term enrichment analyses of RNA-seq data suggests that they reach many readers and may influence our overall understanding of yeast biology. However, GO term lists are not static and are subject to regular updates, with recent releases containing increased numbers of experimentally derived annotations (Fig. 2B). These alterations in GO annotations cause individual gene sets to change in size (Fig. 2C and D). We hypothesize that the prevalence of GO term enrichment coupled with the flux in gene set annotations could cause many analyses, especially those published several years ago, to yield different results upon reanalysis.

**Figure 3.**
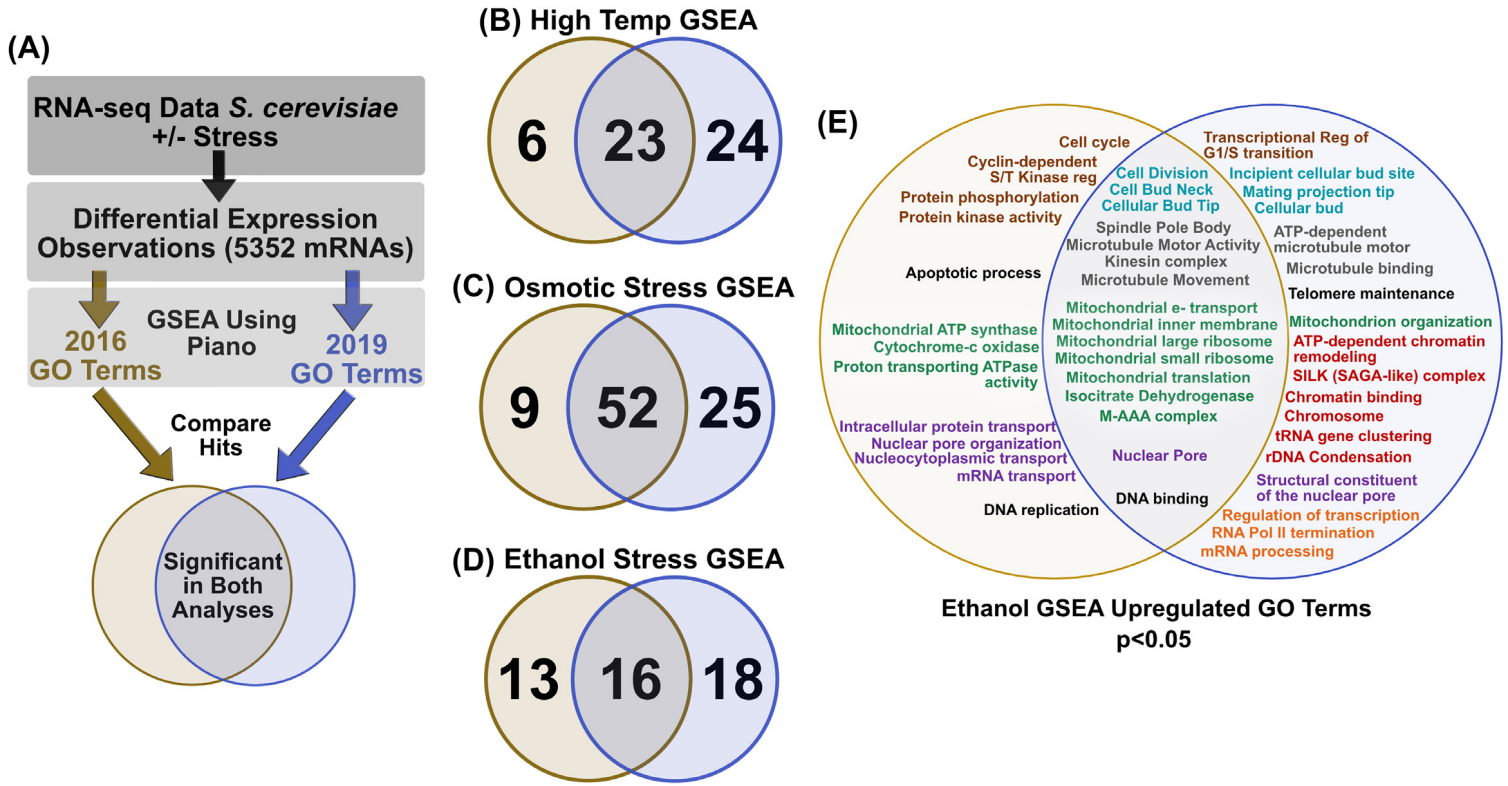
Gene set enrichment analysis results change over time. **(A)** DE data (RNA-seq) from Lahtvee 2016 was analyzed for enriched gene sets among either 2016 GO terms (gold) or 2019 GO terms (blue). Analysis utilized the piano R-package and included gene sets with 2 to 400 genes. **(B–D)** Significantly enriched GO term overlap is shown between the two analyses for gene sets that were upregulated**(E)**. Upregulated GO terms for ethanol stress were grouped using text color based on similarity. GO terms shown in black were not placed into groups.

To test how much GO term enrichment results change over time, we utilized the strategy shown in Fig. 3A. Briefly, GSEA was run to assess the upregulated GO terms from a published dataset that investigated stress due to elevated temperature, high osmolarity or ethanol exposure for *S. cerevisiae* (Lahtvee *et al*. 2017). These analyses utilized the same version of the R-package piano (Väremo, Nielsen and Nookaew 2013), the same published DE data, but different GO annotation lists retrieved either from the Ensembl archive for October 2016 or from Ensembl at the time of the analysis (July 2019) (Fig. 3A) (http://www.ensembl.org and http://oct2016.archive.ensembl.org/). This analysis found that of the GO terms present in both 2016 and 2019, several were significantly upregulated in only one of the GSEA analyses (Fig. 3B–D).

To compare these results more thoroughly, the overlapping and discordant GO terms for ethanol stress are shown in Fig. 3E. GSEA results from the ethanol analyses suggest that some similar GO terms were enriched (e.g. mitochondrial terms shown in green) despite changes in GO annotations. However, some of the enriched GO terms from the 2019 analysis strengthen potential hypotheses compared to 2016 (e.g. cellular budding is perturbed [light blue]) or suggest new hypotheses altogether (e.g. chromatin organization genes are upregulated [highlighted in red]) (Fig. 3E). These changes in GSEA results after less than 3 years suggest that reanalysis of GO term enrichment is a viable method for generating new hypotheses from existing RNA-seq data. These reanalyses may have particular value if run before designing a new experiment based on published results, or when attempting to test a hypothesis using a broad analysis of several datasets. GO term enrichment is one example of how new scientific data can change the results of transcriptome analyses. The findings of this section indicate that GO term analyses published today represent the ‘best current information’ on gene functional enrichment. While these analyses may identify a surprising new direction for your research, it is important to analyze the underlying individual gene data and scrutinize the boundaries of each gene set. Furthermore, it is important to keep in mind when reading publications with these data types that the results presented are limited by the annotations that are available at the time of publication.

## SECTION 2: NOVEL ANALYSES USING GENE METRICS INSTEAD OF GENE FUNCTIONS

**Figure 4.**
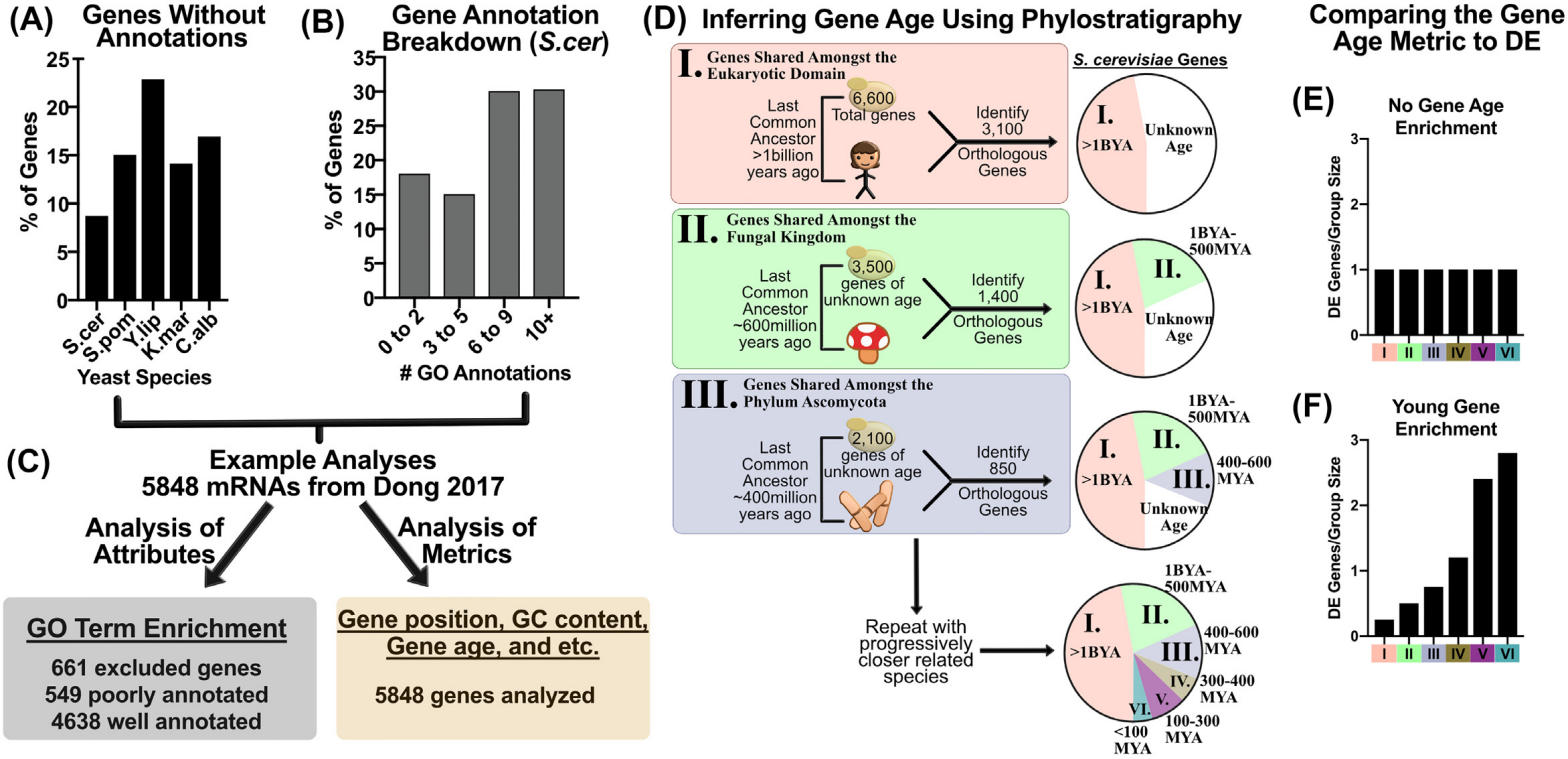
RNA-seq analyses based on gene metrics, like Phylo-DE, enable analysis of all measured mRNAs. **(A)** Genes lacking GO term annotations for commonly studied yeast species. **(B)** GO annotations are shown from Ensembl biomart using current GO terms (2019). **(C)** Examples of RNA-seq data analysis using attributes like GO Terms (gray) compared to analyses using metrics like gene age (beige). **(D)** The phylostrigraphy methodology is shown, gene age is inferred by the identification of an orthologous gene in organisms with decreasing last common ancestor evolutionary distance. **(E)** Hypothetical Phylo-DE analysis showing no relationship between gene age and DE. **(F)** Hypothetical Phylo-DE analysis showing enrichment for young gene DE.

Analyses of functional changes, like GO enrichment, in an RNA-seq dataset are useful for generating hypotheses. However, these techniques are unable to include some genes due to our incomplete understanding of yeast gene functions. For example, genes of unknown function represent 9–23%, or 482–1476 protein-coding genes among the yeast species listed in Fig. 4A. Furthermore, an additional subset of ‘known genes’ have only one general annotation such as ‘cytoplasm’ or ‘mitochondria’ (Fig. 4B). Dismissing these genes as uninteresting would be convenient, but the number of unknown genes has decreased steadily over time (Fig. 2B), suggesting that many of these genes may have functions that we have yet to characterize. In this section, we discuss analyses that compare the DE of all genes using quantifiable traits (called metrics herein) that are available or can be generated for all genes (Fig. 4C). These observations can at first seem rudimentary, but strong associations in large datasets can act as anchor points, from which other analyses can radiate. Furthermore, as new metrics are created and shown to be valuable, data analysts benefit by adding new ideas to their knowledge base.

Relationships between gene expression and gene metrics, such as chromosomal location (Chen *et al*. 2013), presence of promoter sequence elements (Bregman *et al*. 2011; Espinar *et al*. 2018), transcription factor binding proximity (Pang *et al*. 2017; Lage *et al*. 2019; Zara *et al*. 2019) and codon usage (Neymotin, Ettorre and Gresham 2016), have been previously described for *S. cerevisiae*. Consider that these metrics needed to be discovered and that new metrics continue to be recorded, opening up new possibilities for data analysis projects. Rather than testing every metric imaginable for associations with your DE dataset, it might be worthwhile to assess whether an interesting metric has shown itself already. For example, do non-RNA-seq experimental observations or anomalies in your RNA-seq analysis suggest a metric to analyze? An example of validating a metric that was observed from experimental data was reported recently (Espinar *et al*. 2018). In this report, experimental evidence suggested that variations in promoter architecture (TATA-presence) and Open Reading Frame (ORF) sequences (codon usage) influence transcript levels. These findings brought quantifiable metrics to the authors’ attention, which inspired reanalysis of archived RNA-seq DE data in relation to ORF sequence and promoter architecture (Espinar *et al*. 2018).

Other analyses of metrics can be tested without prior observation of an anomaly in a dataset by querying existing databases. For example, differentially expressed genes can be compared to transcription factor binding/expression observations by querying the YEASTRACT database (http://www.yeastract.com/index.php) (Teixeira *et al*. 2017). Recent works have used YEASTRACT to associate transcriptomic changes with specific transcription factors to support a transcriptomics driven storyline (Pang *et al*. 2017; Lage *et al*. 2019; Zara *et al*. 2019). Database searches like this enable rapid comparison of differentially expressed genes to an existing metric and allow the user to convert a list of differentially expressed genes into a potential hypothesis. Notably, relatively few metrics have been converted into web-based tools, which is an obstacle to hypothesis generation and an opportunity for the analyst to discover something novel.

Another example of analysis of a metric comes from our own work focused on testing a perceived anomaly in the stress responses of various yeast species. This analysis found a relationship between evolutionary gene age and DE in response to stress adaptation (Doughty *et al*. 2019). In this work, genes that are conserved between yeast species were found to be statistically rare among stress responsive genes. This result was viewed as an anomaly and led to the hypothesis that evolutionary conservation of a gene might correlate with its likelihood to be differentially expressed in response to stress. Fig. 4D shows the method used to infer the evolutionary age of genes using orthology inference software like OrthoFinder (Emms and Kelly 2015) or OrthoMCL (Li, Stoeckert and Roos 2003) in stepwise orthology searches. This process was repeated to stratify each gene into a single group based on the predicted evolutionary gene age (termed phylostratigraphy) (Domazet-Lošo, Brajković and Tautz 2007), thus establishing a metric, which was compared to newly created and archived DE data. This analysis showed that gene age correlated with stress DE, which was the main conclusion of the work (Doughty *et al*. 2019).

In each of the cases described, quantifiable gene metrics were used to extract information that compliments traditional differential gene expression from RNA-seq. Analysis of metrics in relation to RNA-seq enables analysts to test hypotheses or add robustness to observations from their field of interest. Furthermore, once a metric is established, it can be tested for a relationship with new or archived data with relative ease. Similar to our findings in section 1 about GO enrichment, analysis of metrics is limited by the metrics that have been previously described. This is both a limitation to the number of available plug and play analyses and an opportunity to observe new metrics and create new tools to benefit future works.

## SECTION 3: USING RNA-seq DATA TO IDENTIFY AND ANALYZE lncRNAs

Sections 1 and 2 of this review focused on extracting information from RNA-seq to discover additional information about annotated protein coding mRNAs. Analyses of mRNAs have been used extensively to identify correlations and enrichments that help to unravel biological phenomena. Notably, the focus on protein-coding genes in many analyses often excludes non-coding RNAs. Non-coding RNAs include small RNAs (e.g. small nuclear RNAs), tRNAs, rRNAs and long non-coding RNAs (lncRNAs) (Parker *et al*. 2018). In this section, we investigate opportunities to detect and analyze lncRNAs in yeast RNA-seq datasets. We focus on lncRNAs since many are poly-adenylated and capped (Tuck and Tollervey 2013) and are, therefore, commonly isolated and measured in RNA-sequencing runs (Wery *et al*. 2016). Over time, scientists have found functions for some of these lncRNA species, which could present opportunities for extracting additional information from your RNA-seq data.

**Figure 5.**
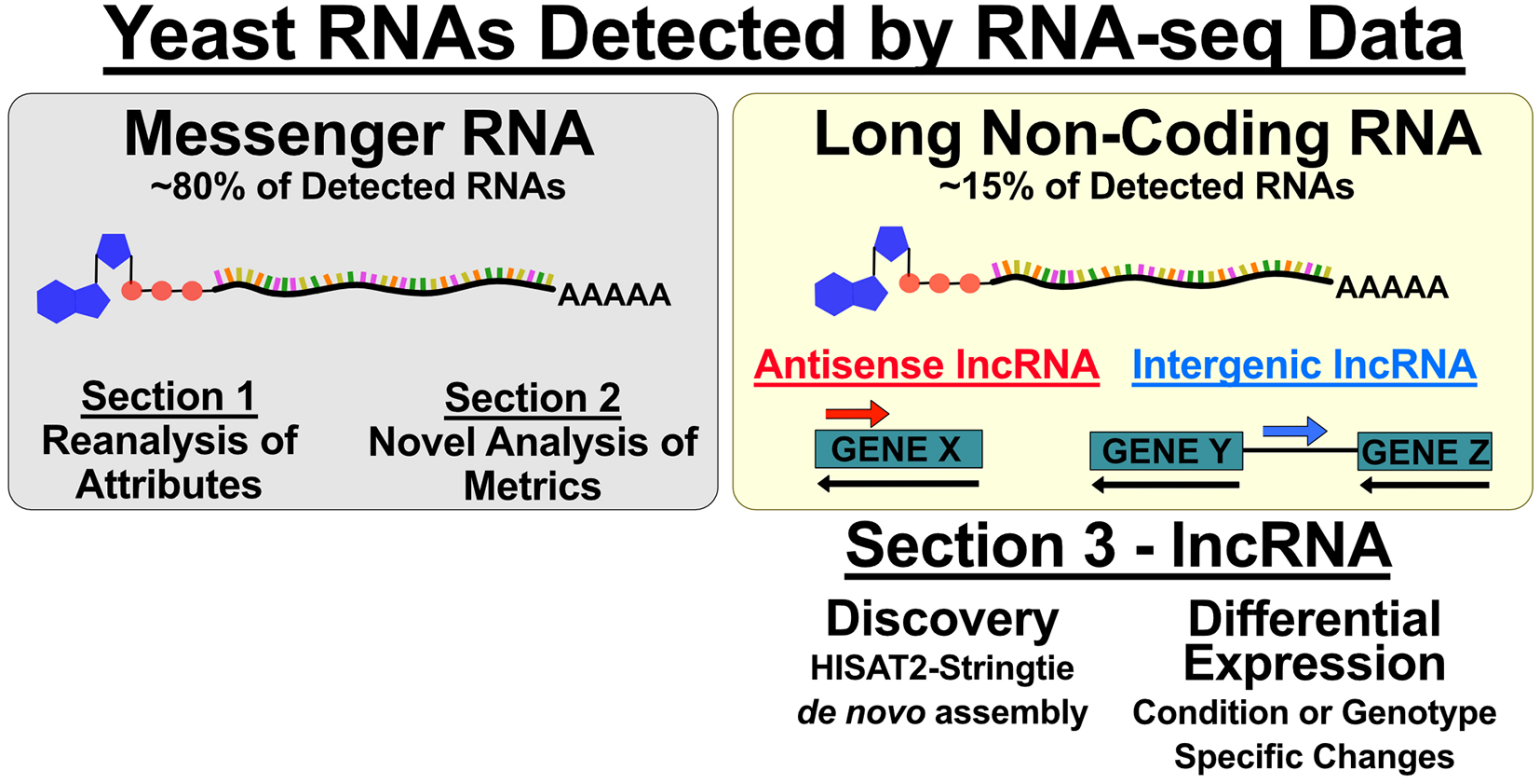
Long non-coding RNAs (lncRNAs) are detected by RNA-seq. Poly-A RNAs are commonly isolated for RNA-seq followed by mRNA analysis (left). Long non-coding RNAs are also polyadenylated and are, therefore, measured by many RNA-seq analyses (right). This provides researchers the opportunity to discover and assess the expression of lncRNAs from existing RNA-seq data.

David *et al*. 2006 published a tiling array for transcriptional activity in *S. cerevisiae* and found that 16% of transcribed bases belonged to unannotated transcripts (referred to as lncRNAs in the section). In total, 126 unannotated transcripts were found among intergenic regions (distinct from 5’ or 3’ UTRs) and 402 were found antisense to known ORFs (David *et al*. 2006) (Fig. 5). Later work found *S. cerevisiae* expressed 847 stable unannotated long non-coding RNA species using 5’ RACE (Xu *et al*. 2009). Further works used RNA-seq to identify lncRNAs and found 23% (biofilm samples) and 9.5% (single cell RNA-seq) of detected *S. cerevisiae* RNA species were lncRNAs (Wilkinson *et al*. 2018; Nadal-Ribelles *et al*. 2019). To clarify, these numbers pertain to detected species number and that lncRNAs represent a small percentage of the total reads from a yeast RNA-seq dataset (Yassour *et al*. 2010). Many lncRNAs are expressed at low copy number, which might be expected as some have been shown to participate in transcriptional downregulation (Martens, Laprade and Winston 2004) or upregulation (Nadal-Ribelles *et al*. 2014) of a single ORF by altering transcription factor binding. More abundant lncRNAs might participate in protein-RNA complexes, like telomerase (Cusanelli and Chartrand 2015) or might contribute to RNA interference in RNAi+ yeasts like *Schizosaccharomyces pombe* (Shah *et al*. 2014).

In addition to the identified lncRNAs in *S. cerevisiae*, hundreds of lncRNAs have been identified in *S. pombe* (Till, Mach and Mach-Aigner 2018), *Fusarium graminearium* (Kim *et al*. 2018), *Neurospora crassa* (Cemel *et al*. 2017) and *Pichia pastorus* (Sun *et al*. 2019). However, at the time of this publication several yeast species have yet to be analyzed for the presence and extent of lncRNA expression, including *Yarrowia lipolytica* and *Kluyveromyces marxianus. De novo* lncRNA annotation is possible with RNA-seq data (Atkinson, Marguerat and Bähler 2012; Wery *et al*. 2016) and existing open access tools (e.g. HISAT2-Stringtie) (Kim, Langmead and Salzberg 2015; Pertea *et al*. 2015), and could provide an initial map of putative lncRNAs for unannotated species. These newly annotated lncRNAs could be of broad interest if they are conserved among several yeast species, as was shown for several lncRNAs (David *et al*. 2006). Additionally, expression of lncRNAs datasets could be condition specific and conserved across multiple yeast species (Yassour *et al*. 2010).

The recent work in this field shows that RNA-seq data analysis is not only a powerful tool for understanding transcriptomic trends occurring among mRNAs (section 1 and 2). While yeast lncRNA research is still in its early stages, the mechanisms of action of specific lncRNAs have come to light through recent work (Till, Mach and Mach-Aigner 2018). In the future, continued single transcript and transcriptome-wide analyses of these species may unlock further insights. Notably, due to their exclusion from most analyses and their presence in poly-A enriched RNA-seq experiments, lncRNAs may allow novel findings to be extracted from archived datasets. Furthermore, an analysis plan based on archived data might speed up the generation of hypotheses about transcriptome-wide trends in lncRNA expression. We suspect that the rate limiting step to unraveling the impact of more lncRNAs on cellular function may be the generation of creative hypotheses born from, supported by, and/or tested with RNA-seq analysis.

## OUTLOOK

In this work, we discuss analyses that can enable information of scientific value to be extracted from RNA-seq datasets. As scientific inquiry continues to improve the overall understanding of yeast biology, new data analysis concepts become apparent. This phenomenon suggests that it is unlikely that a single paper, published at a single point in time, can exhaust the scientific value of a dataset. We note that free access to archived data, tutorials and programs creates the opportunity for more students, data analysts and even professors to ask creative questions. This democratization of data and programs enables more scientists to grapple with the most important challenge in data analysis: generating more accurate hypotheses and ideas that get to the core of the phenomena in question.
